# Age-Related Differences and Cognitive Correlates of Self-Reported and Direct Navigation Performance: The Effect of Real and Virtual Test Conditions Manipulation

**DOI:** 10.3389/fpsyg.2015.02034

**Published:** 2016-01-25

**Authors:** Mathieu Taillade, Bernard N'Kaoua, Hélène Sauzéon

**Affiliations:** ^1^Laboratory of “Disability and Nervous system”, Collège Sciences du vivant et de la Santé, University of BordeauxFrance; ^2^Phoenix Team, Inria Center of BordeauxInria, Talence, France

**Keywords:** aging, spatial learning, direct and self-reported navigation performance, real and virtual tests

## Abstract

The present study investigated the effect of aging on direct navigation measures and self-reported ones according to the real-virtual test manipulation. Navigation (wayfinding tasks) and spatial memory (paper-pencil tasks) performances, obtained either in real-world or in virtual-laboratory test conditions, were compared between young (*n* = 32) and older (*n* = 32) adults who had self-rated their everyday navigation behavior (SBSOD scale). Real age-related differences were observed in navigation tasks as well as in paper-pencil tasks, which investigated spatial learning relative to the distinction between survey-route knowledge. The manipulation of test conditions (real vs. virtual) did not change these age-related differences, which are mostly explained by age-related decline in both spatial abilities and executive functioning (measured with neuropsychological tests). In contrast, elderly adults did not differ from young adults in their self-reporting relative to everyday navigation, suggesting some underestimation of navigation difficulties by elderly adults. Also, spatial abilities in young participants had a mediating effect on the relations between actual and self-reported navigation performance, but not for older participants. So, it is assumed that the older adults carried out the navigation task with fewer available spatial abilities compared to young adults, resulting in inaccurate self-estimates.

## Introduction

Spatial orientation and wayfinding ability in familiar and unfamiliar environments are critical for daily functioning and independent living. Even in advanced age, many elderly people remain able to perform self-orientation tasks efficiently in familiar environments but they often experience some difficulties when the environment is unfamiliar (e.g., Kirasic, 1989; Willis, 1991; Baroni and De Beni, 1995). When an age-related difference is reported, it is widely explained as the result of age-related decline in small-scale spatial abilities or in the ability to acquire new spatial information (i.e., memory decline) or in the implementation of the strategic navigation behavior (i.e., executive decline) required in unknown large-scale environments (for reviews, Moffat, 2009; Klencklen et al., 2012; Lithfous et al., 2013). Such interpretations are mainly claimed in laboratory-based studies from tasks such as real full-scale maze or virtual full-scale environment (Moffat, 2009; Klencklen et al., 2012; Lithfous et al., 2013). Paradoxically, elderly people self-report few everyday navigation complaints, even when they are specifically consulted about outdoor displacements in unfamiliar or unknown environments (Kirasic et al., 1992; Burns, 1999; De Beni et al., 2006; Taillade et al., 2012, 2013; Borella et al., 2014), Such results indicated no age-difference on self-reported large-scale spatial measures. Therefore, the purpose of this aging study was to address the mismatch on the one hand, between direct spatial measures as they are taken in a real situation or laboratory situation (i.e., real vs. virtual environment manipulation) and on the other hand, between direct and self-reported spatial measures (i.e., relationships between direct and self-reported measures).

Concerning the direct measurements of navigational processing, first a decline associated with aging has been reported in navigational and wayfinding performance, for instance in real mazes (Newman and Kaszniak, 2000) or virtual mazes (e.g., Moffat and Resnick, 2002; Antonova et al., 2009; Head and Isom, 2010) or even in more naturalistic virtual environments such as urban districts or museum (Lövdén et al., 2005; Iaria et al., 2009). Secondly, an age-related decline in spatial learning and memory performance has been reported for large-scale environments (for review, see Moffat, 2009; Klencklen et al., 2012). Indeed, age-related difficulties in acquiring landmark, route and survey knowledge has been observed in laboratory-tasks irrespective of the level of spatial representations according to the Landmark-Route-Survey model by Siegel and White (1975). For instance, this difficulty is observed in full-size mazes composed of hallways (Kalia et al., 2008) or in virtual maze (e.g., Moffat et al., 2001; Head and Isom, 2010) or in more realistic virtual-environments (virtual museum: Lövdén et al., 2005; virtual house: Meulenbroek et al., 2004; virtual town: Iaria et al., 2009; Head and Isom, 2010; Jansen et al., 2010; Gyselinck et al., 2013). Thus, when laboratory tasks are used, older adults exhibit difficulties in acquiring spatial knowledge relative to perceived details, performed paths and the layout of environments. However, such a conclusion cannot be generalized to results from studies using real-world environments. Indeed, some of them reported similar age-related differences to those observed with laboratory-tasks (Lipman and Caplan, 1992; Wilkniss et al., 1997; Kirasic, 2000) while some other failed to find age-related differences (Kirasic, 1991; Meneghetti et al., 2012). Several complementary methodological explanations have been advanced for these inconsistent results. First, some authors stressed the role of environment familiarity as a critical factor on the occurrence of age differences, where the age differences decrease with the increasing familiarity of the environment (e.g., Kirasic, 1989; Willis, 1991). Thus, older adults are able to cope effectively with tasks requiring navigation or self-orientation in familiar environments. Another factor proposed is the ecological value of the tasks used (the extent of similarity with everyday life situations), where older adults are presumed able to deal with spatial deficits in navigating new environments using efficiently their long years of experience in orienting themselves (e.g., Willis, 1991; De Beni et al., 2006). In other words, the elderly people exhibit a dramatic decline during more abstract or laboratory tests, but their peformances are adequate or nearly normal in more natural tasks, as claimed by De Beni et al. (2006). This last assumption might benefit from insights provided by the studies comparing age-related differences in navigational processing according to real vs. virtual environment manipulations. Indeed, a decrease of age-differences in real condition compared to virtual one is supportive of experience-based coping strategies that are expressed only in conditions of real-world everyday tasks.

Only three studies have addressed the age-related differences in navigational processing according to real vs. virtual environment manipulations. First, Kalia et al. (2008) have reported comparable age effects in real or virtual conditions on several spatial measurements derived from indoor environment composed of neutral hallways. Secondly, Kalova et al. (2005) have compared virtual and real-world navigation tasks by using a human version of the Morris water-maze (i.e., a circular arena equipped with a computerized tracking system) and asked elderly participants (elderly and patients with Alzheimer's disease but without signs of dementia) to locate one or more unmarked goals using the arena geometry, their starting position and/or cues on the arena wall. The results indicated that navigation performed in the real world and that done using a similar computer version yielded comparable results. Finally, using hospital environments, Cushman et al. (2008) have found results consistent with those of Kalova et al. (2005), and report a strong correlation (*r* = 0.73) between the performance of spatial tasks carried out in the real lobby of a hospital and in a similar virtual-world situation. Taken together, this suggests equivalent age-related differences in the real and virtual-laboratory tests that are not in favor of intact real-world everyday tasks performance with aging due to the use of experience-based strategies in older adults. Such a conclusion would be hasty given that the paucity of studies and the ecological value of tasks used (maze, hallways for two studies among the three performed). For this reason, we proposed to compare the age-related differences in navigational processing and spatial learning from everyday tasks performed either in real urban environment or in virtual urban environment. Additionally, we proposed to study whether cognitive mediators such as spatial abilities, memory, and/or executive functioning play the same role in the age-related difference in the direct navigation performances, in virtual and real conditions. Indeed, considerable laboratory-based evidence has supported the hypothesis that age-related differences in navigation behavior are widely mediated by small-scale spatial abilities (Meneghetti et al., 2011; Gyselinck et al., 2013) and memory decline, as well as age-related decline in executive functioning (e.g., Taillade et al., 2012, 2013; for review, Moffat, 2009; Klencklen et al., 2012; Lithfous et al., 2013). If the cognitive mediators are differently involved in age-related differences according to the real- virtual manipulation, this can provide relevant insights explaining the discrepant results between the laboratory-based studies and the real world-based studies. From the overall data, more the real-virtual manipulations will be studied, the more it will be possible to understand the results differences between the laboratory based- studies and the real-world based studies.

Regarding the self-reported measurements of navigational processing, older adults tend to judge their sense of direction as well as navigational performance more positively than the younger counterpart. Indeed, elderly people self-report few everyday navigation complaints even, when they are specifically consulted about outdoor displacements in unfamiliar or unknown environments, like those proposed in self-questionnaires investigating everyday spatial disorientation (Kirasic et al., 1992; Burns, 1999; De Beni et al., 2006; Taillade et al., 2012, 2013; Borella et al., 2014). This result can be interpreted in two manners. Firstly, the elderly people exhibit adequate or nearly normal performance in real world or everyday tasks and thus, no navigation complaint is expressed. This fits with the view of spared performance in a real-world setting contrasting with a dramatic decline in more abstract or laboratory tests as proposed by De Beni et al. (2006). Second, the lack of navigational complaint in the older people indicates that their self-reporting is inconsistent with their actual navigation problems (Kirasic et al., 1992; Taillade et al., 2012, 2013). For instance, Taillade et al. (2012) reported age-related differences in the wayfinding scores as in spatial learning measurements, contrasted with unchanged self-reported navigation performance by age. And, the direct wayfinding performance was strongly related to the self-reported navigation performance, only when the age factor was partialled out. This supports the idea that the older adults were less aware of, or less accurate in their judgments of navigational functioning than the young ones, probably in relation to age-related metacognitive difficulties which are well known in the cognitive aging field (e.g., Vanderhill et al., 2010). However, more evidence is required to support this statement, and notably to demonstrate that this relationship loss with age has not been confused with a possible laboratory-test effect. Indeed, Hegarty et al. (2006) have demonstrated in young adults that measurements of small-scale spatial abilities (such as those explored by the Mental Rotation Test of Vandenberg and Kuse, 1978) were relatively more predictive of spatial learning of naturalistic large scale environment from VR based media than learning from direct experience, whereas self-reported sense of direction (probed by the SBSOD) was more predictive of learning from direct experience than from VR-based media. In other words, the weight of variables predicting navigational performance is influenced by the manipulation of the real vs. virtual condition. This finding may indicate that age-related loss of relationship between the direct and self-reported navigation performance could be due to the increased involvement of spatial abilities in virtual-laboratory tests compared to real-world tests. So, this was a motivation to explore the mediating role of cognitive predictors of decline related to aging as a possible explanation of age-related differences on navigational measures and of the age-related loss of relations between direct and self-reported navigational measures.

Based on the above reports, we first proposed a direct comparison of wayfinding and spatial learning performances in an urban district, either through direct experience (real conditions) or with a more abstract experience using a virtual replica (virtual-laboratory test). Secondly, we explored the mediating role of cognitive predictors (small scale spatial abilities, memory, and executive functioning) on navigational measure according to the real-laboratory test manipulation. Finally, we proposed to assess the relationship between the actual navigation measurements and the self-reported ones, according to the age group and according to mediating effects of cognitive predictors (small scale spatial abilities, memory, and executive functioning).

## Method

### Participants

In all, we recruited 64 adults—32 young healthy adults (mean age = 22.65; *SD* = 3.29) and 32 older adults (mean age = 68.58; *SD* = 6.13). All of the participants were volunteers, native French speakers, right-handed, and without experience of the studied district. Indeed, all the participants were asked their familiarity with the district used in the experiment and the participants familiar with the environment were excluded. From a general questionnaire, they also reported that they were healthy and without any visual, neurological or psychiatric disorder. Young adults were recruited at the University of Bordeaux and the older adults were recruited from a “Senior” University in Bordeaux (“Université du Temps Libre”). Older adults underwent the MMSE (Folstein et al., 1975) as an exclusion criterion (exclusion for a score < 27). All of the subjects under virtual-laboratory condition had to complete a French version of the Simulator Sickness Questionnaire (SSQ; Kennedy et al., 1994) immediately after the learning phase. This questionnaire measures the severity of sickness induced by 3D simulators. Participants also had to rate their own technology experience with computers and computer games (New Technology of Information and Communication-NTIC questionnaire, Moffat et al., 2001). There were no significant inter-group differences in SSQ score (*p* > 0.05) and education level (*p* > 0.05) (Table 1). An age effect was reported for the NTIC score [*t*_(30)_ = 4.03; *p* < 0.001].

**Table 1:** **Demographic characteristics of the participants**.

**Environment of learning and restitution**	**Young group**		**Older group**		**ANOVA results/Group comparaisons**
	**RE^a^ Mean (*SD*)**	**VE^b^Mean (*SD*)**	**RE Mean (*SD*)**	**VE Mean (*SD*)**	***Age effect/Environment effect/Age × Environment effect***
Male/Female	8/8	8/8	8/8	7/9	
Age	22.06 (2.70)	23.25 (3.70)	66.06 (4.60)	70.81 (6.60)	*Age effect: F*_(1, 60)_ = 1489.37; *p* < 0.0001; η^2^ = 1.00 *Environment effect: F*_(1, 60)_ = 0.21, *p* = 0.65; ns *Age* × *Environment effect: F*_(1, 60)_ = 1.04, *p* = 0.31; ns
MMSE	–	–	29.25 (0.68)	29.125 (1.03)	*Environment effect*: *t*_(30)_ = 1.14; *p* = 0.26; ns
NTIC	–	15.25 (3.55)	–	9.68 (4.25)	*Age effect: t*_(30)_ = −4.02; *p* < 0.001; η^2^ = 0.98
Educational level	14.75 (1.48)	13.56 (1.90)	12.87 (3.06)	14.37 (3.86)	*Age effect: F*_(1, 60)_ = 0.59; *p* = 0.44; ns *Environment effect: F*_(1, 60)_ = 0.05, *p* = 0.82; ns *Age × Environment effect: F*_(1, 60)_ = 3.80, *p* = 0.07; ns
SSQ	–	10.52 (16.05)	–	10.52 (18.58)	*Age effect: t*_(30)_ = 1.16; *p* = 0.25; ns

a *RE, Real Environment*;

b *VE, Virtual Environment; SD, Standard Deviation*.

Even if this study does not meet the bio-medical criteria for the CPP-III^1^ assessment, each participant signed a consent form in order to obtain the approval of each participant, as recommended by the CPP-III and the Helsinki convention. This document explains the process and the reasons for the study and how the behavioral data collected will be used. A written informed consent was obtained from each participant. All data were analyzed anonymously.

### Procedure

#### Neuro-cognitive assessment

Each participant undertook a set of neuropsychological tests to assess their cognitive functioning assessing for main cognitive domains: processing speed (SP), spatial abilities (Sp-A), visuo-spatial memory (V-Mem), and executive functioning (Exe-F).

#### Visuo-spatial abilities (Sp-A)

The Sp-A evaluation included the *mental rotation abilities* with The Mental Rotation Test—MRT- (Vandenberg and Kuse, 1978) and the *visuo-spatial working memory* with the Backward Corsi Span Tests (BCS) of the WMS-III (Wechsler, 1997).

#### Visuo-spatial memory (VS-M)

The immediate Visual Reproduction Tests from the Wechsler Memory Scale-III (WMS-III, Wechsler, 1997) and the Benton Visual memory test (Benton, 1965) were used.

#### Executive functioning (Exe-F)

The VS-EF evaluation included *cognitive flexibility* with part B of the Trail Making Test (TMT- B, Reitan, 1992) and *inductive reasoning abilities* with Raven's Matrices Test (standard form; Raven et al., 2003).

All these tests were carried out before and after the spatial learning and navigation tests in real or virtual-laboratory environment conditions and their order were counterbalanced between subjects. For each group, the mean values of all neuropsychological measurements are presented in Table 2.

**Table 2:** **Scores on the neuropsychological assessments: Visuospatial abilities (SP-A), Visuospatial memory (VS-M) and executive functioning (Exe-F)**.

**Environment of learning and restitution**	**Young group**		**Older Group**		**ANOVA results/Group comparaisons**
	**RE^a^**	**VE^b^**	**RE**	**VE**	***Age effect/Environment effect/Age* × *Environment effect***
	**Mean (*SD*)**	**Mean (*SD*)**	**Mean (*SD*)**	**Mean (*SD*)**	
**SP-A**					
Mental Rotation Test	21.19 (7.69)	22.31 (8.36)	8.13 (3.90)	9.13 (6.21)	*Age effect: F*_(1, 60)_ = 60.32; *p* < 0.0001; η^2^ = 1 *Environment effect: F*_(1, 60)_ = 0.39; *p* = 0.53; ns *Age* × *Environment effect: F*_(1, 60)_ = 0.001; *p* = 0.97; ns
Backward Corsi Span Test	8.63 (2.34)	8.25 (1.29)	6.81 (1.52)	7.00 (2.10)	*Age effect: F*_(1, 60)_ = 10.864; *p* < 0.01; η^2^ = 0.92 *Environment effect: F*_(1, 60)_ = 0.04; *p* = 0.84; ns *Age* × *Environment effect: F*_(1, 60)_ = 0.36; *p* = 0.54; ns
**VS-M**					
WMS-III: Immediate Recall	96.50 (14.72)	95.81 (9.18)	86.25 (11.13)	86.63 (12.34)	*Age effect: F*_(1, 60)_ = 10.472; *p* < 0.01; η^2^ = 0.91 *Environment effect: F*_(1, 60)_ = 0.003; *p* = 0.95 ns *Age* × *Environment effect: F*_(1, 60)_ = 0.03; *p* = 0.86; ns
Benton's Visual Recognition Test	14.63 (0.72)	14.19 (1.05)	13.13 (1.31)	13.63 (1.21)	*Age effect: F*_(1, 60)_ = 13.49; *p* < 0.001; η^2^ = 0.97 *Environment effect: F*_(1, 60)_ = 0.01; *p* = 0.91; ns *Age* × *Environment effect: F*_(1, 60)_ = 2.79; *p* < 0.10; ns
**Exe-F**					
TMT B	36.92 (11.84)	35.13 (12.60)	68.53 (25.35)	79.12 (28.41)	*Age effect: F*_(1, 60)_ = 37.85; *p* < 0.0001; η^2^ = 1 *Environment effect: F*_(1, 60)_ = 0.52; *p* = 0.48; ns *Age* × *Environment effect: F*_(1, 60)_ = 1.01; *p* = 0.32; ns
Raven's Matrices Test	55.94 (2.89)	53.32 (6.60)	44.44 (5.18)	45.69 (6.83)	*Age effect: F*_(1, 60)_ = 46.70; *p* < 0.0001; η^2^ = 1. *Environment effect*: *F*_(1, 60)_ = 0.24; *p* = 0.62; ns *Age* × *Environment effect*: *F*_(1, 60)_ = 1.92; *p* = 0.17 ns

a *RE, Real Environment*;

b *VE, Virtual Environment; SD, Standard Deviation*.

For each cognitive domain, a composite score was computed with the Z-score procedure. The three composite scores were used to perform the subsequent correlation analyses.

#### Self-reported navigation performance

The participants completed questionnaires about their *everyday visuo-spatial difficulties a*ccording to the Santa Barbara Sense of Direction Scale (SBSOD, Hegarty et al., 2002). The SBSOD has been demonstrated as mainly related to tasks of spatial knowledge that involve orienting oneself within the environment, such as navigational tasks, rather than tests that involve estimating distances or drawing maps (Hegarty et al., 2002).

#### Direct spatial learning and navigation performance

Within a two-step task including spatial learning of a route followed by a route wayfinding test, two main experimental conditions were manipulated: the Real-world Environment (RE) and Virtual-laboratory Environment (VE).

**Figure 1:**
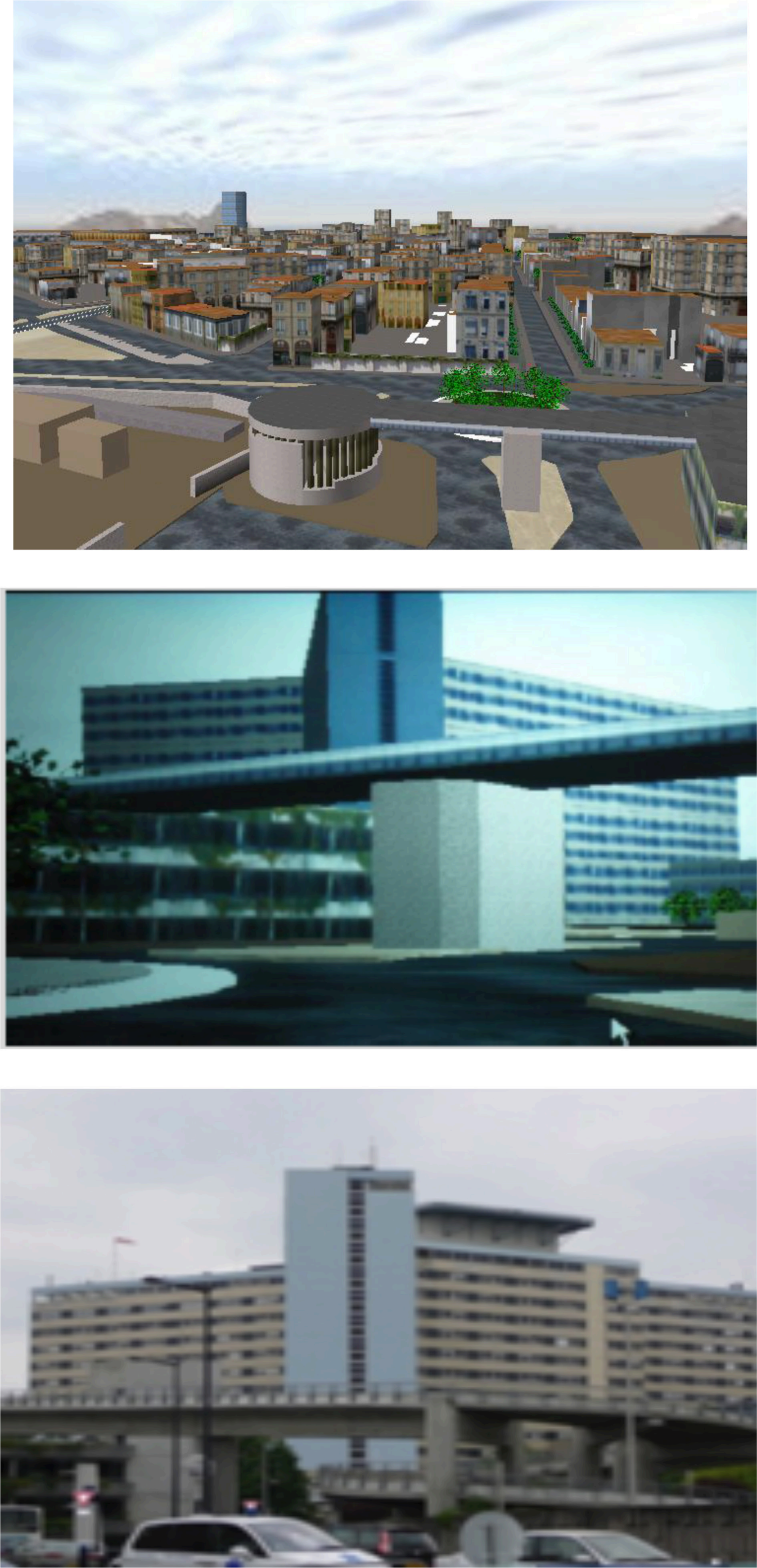
**Aerial and egocentric views from Virtual Environment (Top, adapted from Taillade et al., 2014) and Egocentric view of Real Environment (Bottom) used for navigational task**.

**Figure 2:**
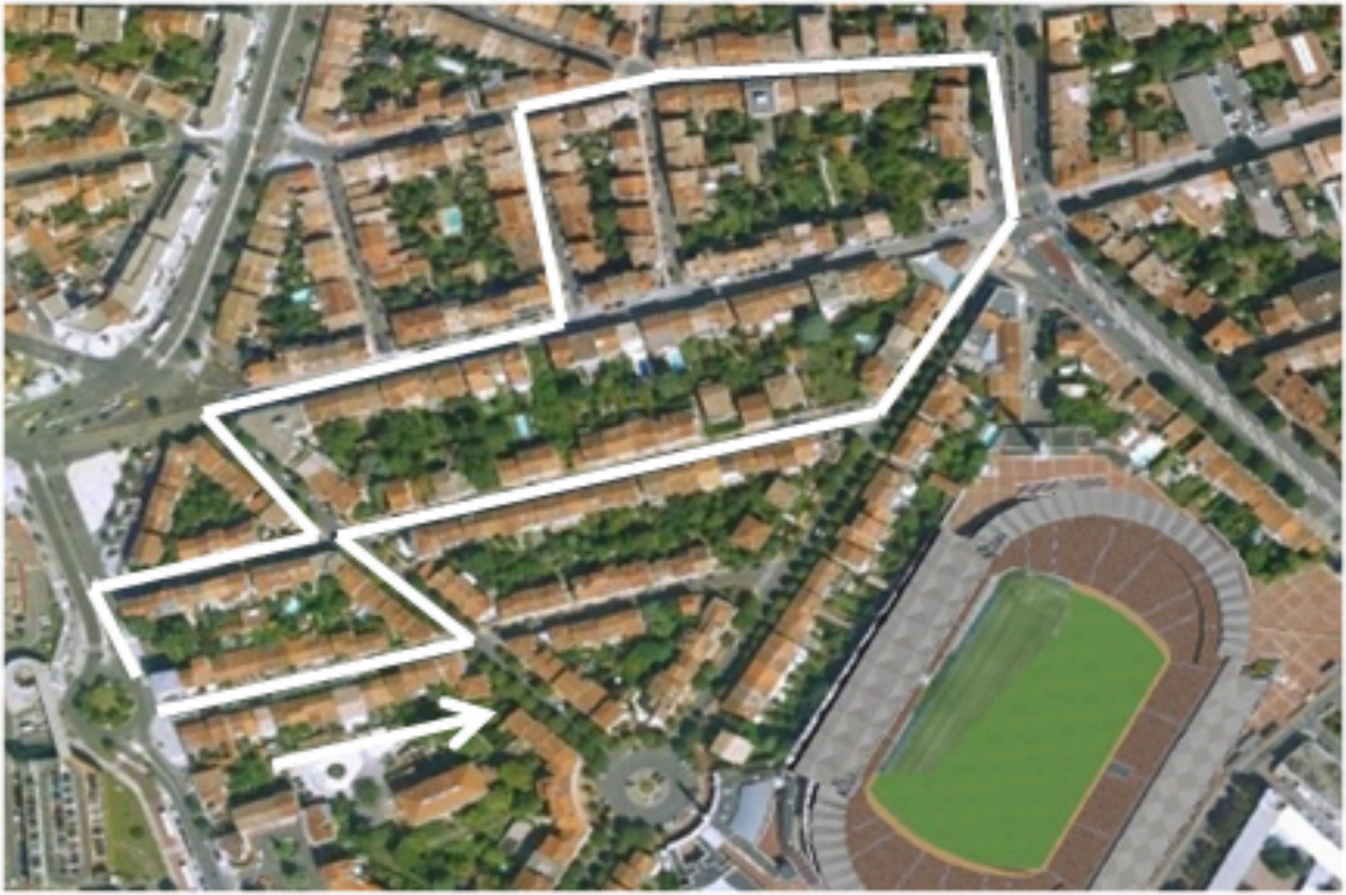
**The route learned by the participants**. Adapted from Taillade et al. (2013, 2014).

The *Real-world Environment* consisted of a district near Bordeaux's hospital while the *Virtual-laboratory environment* was a replica of the real-environment (i.e., the district near Bordeaux's hospital) (Figures 1, 2) created using the Virtools© software. Significant landmarks (signposts, signs, and urban furniture) were included in the VE. Subjects were placed at a distance of 2 from the screen to initiate a semi-immersion effect into the VE. This corresponded to an external 65° horizontal point of view and a 50° vertical point of view for the subjects. VIRTOOLS© produced a 120° internal point of view of the route, which allowed a good perception of the street perspective for navigation with a good level of details. Subjects sat on a standard chair in front of the screen. They displaced with a walking speed of 3 km/h within the VE district according to a subjective view. The walking displacements in the VE were simulated with the manipulation of a joystick for implementing translational and rotational movements that serve the locomotion in the VE. The apparatus used in the VR room was a HP Elite Book 8540 p © personal computer (Intel® Core™ i5 CPU processor in 2.40 GHz, 2 Go RAM) with a NVIDIA© graphics card, a EpsonX EB © 1925 W projector, a 2 × 2-meter screen and a Saitek® X52 Flight System joystick for exploration.

Young and older participants were divided into four groups for assignment to the two experimental conditions (RE vs. VR). A training phase (5 min) took place for the participants assigned to the VE condition, where each participant was trained to navigate in an imaginary VE^2^, to allow the participants to familiarize themselves with the virtual navigation and joystick use and to confirm that none of the participants had major simulator sickness (mostly for the older adults). Before the experiment, participants were asked on their knowledge of district near the hospital. Morevover, this question was re-asked later in the questionnaires.

The procedure was divided into two steps as follows:

1. A learning phase (average 15 min) where the participants learned a route, in the RE or VE; specifically, the participant was brought to the starting point in the real or virtual district and walked a route in order to learn it (a route composed of 10 streets, 13 intersections and 11 changes of direction and 787 meters long^3^) in accordance with the instructions given by the tester. Subjects were instructed to pay attention to the route because they would later undergo testing related to it.
2. A restitution phase, where two kinds of tasks were performed by the participants soon after the learning phase in counter-balanced order.

#### Wayfinding task

The participants were asked to replicate the route that they learned in the same environment used for learning (respectively, RE or VE). To do this, participants were brought to the starting point in the environment and had to recall the route. Wrong turns and stops before deciding to change direction (when the subject stops more than 5 s and looks around) were counted. When a mistake occurred after a stop, both were counted. If the subject made a wrong decision, he was shown the correct direction by the experimenter and allowed to continue on the route. Thus, two scores were calculated from this task during the wayfinding task: the number of direction errors (wrong turns) and the number of stops, as a way to study the use of spatial representation to perform a navigational task (Wallet et al., 2009, 2010, 2011; Lapeyre et al., 2011).

### Spatial memory tasks

- *Picture classification task*, known to be performed well when participants have a well-developed knowledge of the route, was given to the participants (Wallet et al., 2009, 2010, 2011; Lapeyre et al., 2011). The task consisted in chronologically ordering 12 pictures that corresponded to different fields of view of the district encountered along the route during the learning phase. The score is a sequence score: 1 point is given if the photo position corresponds to the absolute correct position in the overall sequence and 0.5 point is given if the position is incorrect but is correctly associated to a backward or forward picture according to the pre-established chronological order. The maximum score is 12. This measure probes the route knowledge built from the path execution into the environment.

*- Map drawing task*, known to be performed well when participants have developed a good spatial cognitive map of the environment (Wallet et al., 2009, 2010, 2011; Lapeyre et al., 2011): the subject is required to draw the route learned on a blank sheet of paper. The drawing has to be made of connected segments, representing the linear locomotion and direction changes. The score is the number of correct directions given from the beginning of the route. The maximum score is 11. Less accurate than measures from bidimensional regressions (Tobler, 1994; Friedman and Kohler, 2003), this measure based on local angles can be seen as a good proxy of survey knowledge level (Lapeyre et al., 2011).

All of the material and procedures are derived from previous studies in the spatial learning and navigation domains (Wallet et al., 2009, 2010, 2011).

### Statistical analyses

To reach the study's aims, two-way ANOVA [2 (Age group: young; old) × 2 (Environment: RE; VR)] analyses on each measure was required. To this end, we have performed preliminary test of normality of collected data (Kolmogorov-Smirnov procedure). This preliminary test revealed that all the data exhibit normal distribution except for the measure of picture classification task (*p* < 0.01). Consequently, we have performed the two-way ANOVA analysis for all the measures except for that from the picture classification task. For this latter case, non-parametric analyses have been performed as recommended for non-normal distribution, with the corrected Mann-Whitney U test comparing conditions relative to age or environment manipulation.

After this set of analyses, several sets of correlation analyses were calculated for assessing the mediating effect of cognitive predictors on age-differences in direct navigation measures, according to the environment condition and also for studying of relationships between the direct and self-reported navigation performance.

## Results

### Neuro-cognitive assessment (Table 2)

Two-way ANOVA [2 (Age group: young; old) × 2 (Environment: RE; VR)] analyses were carried out on each neuropsychological measurement. The results are summarized in Table 2. None of these analyses indicated a simple or interaction effect including the condition assignment factor. In contrast, an age effect was significantly observed in all the dependent measurements relative to the three cognitive domains of interest for navigation behavior. Indeed, the young performed better than their older counterparts for spatial abilities (Sp-A), visuo-spatial memory (V-Mem) and executive functioning (Exe-F) measurements (Table 2).

### Self-reported navigation performance from SBSOD (Table 3)

A two-way ANOVA [2 (Age group: young; old) × 2 (Environment: RE; VR)] analysis failed to find any significant effect. Indeed, SBSOD scores were almost equivalent in both the young and the older groups.

**Table 3:** **Scores of the participants for self-reported (from Santa Barabara Sense of Direction Scale, SBSOD) and direct navigation performance**.

**Environment condition**		**Young group**		**Older group**		***Age effect Environment condition effect Age x Environment condition effect***
		**RE^a^**	**VE^b^**	**RE**	**VE**	
		**Mean (*SD*)**	**Mean (*SD*)**	**Mean (*SD*)**	**Mean (*SD*)**	
Self-reported	SBSOD	53.69 (10.34)	54.63 (13.04)	55.81 (16.43)	52.00 (15.84)	*Age effect*: *F*_(1, 60)_ = 0.005; *p* = 0.94; ns *Environment effect*: *F*_(1, 60)_ = 0.16; *p* = 0.69; ns *Age* × *Environment effect: F*_(1, 60)_ = 0.45; *p* = 0.50; ns
Wayfinding	Errors	3.84 (5.62)	6.25 (7.00)	5.77 (5.61)	14.2 (10.83)	*Age effect*: *F*_(1, 60)_ = 6.15; *p* < 0.05; η^2^ = 0.68 *Environment effect*: *F*_(1, 60)_ = 7.38; *p* < 0.01; η^2^ = 0.77 *Age* × *Environment effect*: *F*_(1, 60)_ = 2.36; *p* = 0.13; ns
	Stops	11.54 (8.43)	16.83 (13.24)	10.10 (9.20)	26.44 (18.19)	*Age effect*: *F*_(1, 60)_ = 1.16; *p* = 0.21; ns *Environment effect*: *F*_(1, 60)_ = 11.31; *p* < 0.01; η^2^ = 0.93 Age × *Environment effect*: *F*_(1, 60)_ = 2.95; *p* = 0.09; η^2^ = 0.38
Spatial memory	Map	36.93 (20.85)	32.38 (19.90)	57.38 (20.85)	57.95 (25.03)	*Age effect*: *F*_(1, 60)_ = 3.26; *p* = 0.08; ns *Environment effect*: *F*_(1, 60)_ = 7.48; *p* < 0.01; η^2^ = 0.78 *Age* × *Environment effect*: *F*_(1, 60)_ = 0.30; *p* = 0.86; ns
	Picture	14.77 (12.37)	27.27 (21.51)	22.73 (13.69)	39.20 (26.29)	*Age effect (Mann-Whitney U test)*: *z* = −2.21; *p* < 0.03 *Environment effect (Mann-Whitney U test)*: *z* = −0.77; *p* = 0.44; ns

a *RE, Real Environment*;

b *VE, Virtual Environment; SD, Standard Deviation*.

### Direct spatial learning and navigation performance (Table 3)

Two-way ANOVA [2 (Age group: young; old) × 2 (Environment test: RE; VR)] analyses were carried out on each spatial learning and navigation measurement, except for the picture classification task, for which we used the Mann-Whitney test.

#### For the wayfinding task (Table 3)

The wayfinding errors were higher in the older group than in the young one [*F*_(1, 60)_ = 6.15; *p* < 0.05; η^2^ = 0.68]. Also, they were higher in the VE conditions than in the RE ones [*F*_(1, 60)_ = 7.38; *p* < 0.01; η^2^ = 0.77] but the interaction effect between the age and the environment test factors was not significant [*F*_(1, 60)_ = 2.36; *p* = 0.13; ns]. Also, the wayfinding stops were higher in the VE test than in the RE test conditions [*F*_(1, 60)_ = 11.31; *p* < 0.01; η^2^ = 0.93]. So, no other effect including the age variable was significant (*p* > 0.05).

#### For the spatial memory tests (Table 3)

Regarding the picture classification, a performance superiority of young participants compared with older participants was observed [Mann-Whitney U test; *p* < 0.05]. No other effect was significant (*p* > 0.05). Similarly, for the map drawing task, younger participants exhibited better performances than older participants [*F*_(1, 60)_ = 15.93; *p* < 0.001; η^2^ = 0.98]). By contrast, no other effect including the environment factor was significant (*p* > 0.50).

### Mediating effect of cognitive predictors on age-differences in direct navigation measures according to the environment condition (Table 4)

To assess the relations between direct navigation measurements and the other measures collected, for each environment condition a composite Z-score (labeled “Navigation score”—Znav), including the wayfinding errors and the two spatial memory measures (for which an age effect occurred) was computed.

**Table 4:** **Correlations between the Znav composite score and age before and after the controlling of each one of neurocognitive composite factors (SP-A, Small scale Visuospatial abilities; V-Mem, Visuospatial Memory; Exe-F, Executive functioning)**.

**Correlations**			**Correlations partialled out for**		
		**Age**	**SP-A**	**V-Mem**	**Exe-F**
VE	*Znav r*	**0.55**	0.33	**0.52**	0.32
	*p*	**0.001**	0.07	**0.003**	0.08
RE	*Znav r*	**0.46**	0.23	**0.53**	0.18
	*p*	**0.007**	0.20	**0.002**	0.32

*Values in bold reached the significance*.

From this, two sets of correlation analyses were carried out. The first one addressed the relationships between the Navigation score and age variable for each environment condition (Virtual and Real) (Pearson's correlations). The second set addressed the possible mediating effects of the three cognitive domains studied (Sp-A; V-Mem; Exe-F) by partial correlations procedures (Table 4).

*For the virtual-based navigation score*, its correlation with the age variable was 0.55 (*p* < 0.001). After the neurocognitive scores were partialled out, the *r*-value of this correlation was: 0.33 (*p* > 0.05) for the Sp-A score; 0.52 (*p* < 0.01) for the V-Mem score and 0.32 (*p* > 0.05) for the Exe-F score.

*For the real-based navigation score*, its correlation with the age variable was 0.46 (*p* < 0.01). The *r*-value of this correlation controlled for each neurocognitive score was: 0.23 (*p* > 0.05) for the Sp-A score; 0.53 (*p* < 0.01) for the V-Mem score and 0.18 (*p* > 0.05) for the Exe-F score.

### Relationship between direct and self-reported navigation measures for young and old participants (**Table 5**)

Specific correlation analyses were carried out to address the relationships between the direct and the self-reported navigation measurements for each group (young vs. old participants) (Pearson's correlations) irrespective of the environment condition (real and virtual conditions) since environment conditions did not change age-related differences. We have firstly calculated the correlation between the global navigation measure (Znav score) and the self-reported navigation measure (SBSOD score) for each age group but no significant results were obtained (Young group: *r* = 0.15; *p* > 0. 05; Old group: *r* = 0.23; *p* > 0.05). Also, none mediating effect was significantly observed for the three cognitive domains (Young group: Sp-A: *r* = −0.03; *p* > 0.05; V-Mem: *r* = 0.10; *p* > 0.05; Exe-F: *r* = 0.15; *p* > 0.05; old group: Sp-A: *r* = 0.23; *p* > 0.05; V-Mem: *r* = 0.23; *p* > 0.05; Exe-F: *r* = 0.23; *p* > 0.05). These non-significant results were not surprising in light of studies in young participants revealing that self-reported sense of direction (i.e., SBSOD score) is more related to direct navigation performance or survey knowledge-based scores than to route knowledge-based scores (e.g., Hegarty et al., 2002). Given this, correlation were performed between the self-reported navigation measure (SBSOD score) and each one of the direct navigation measures (wayfinding scores and spatial memory scores). Table 5 presents all the correlations results. Also, possible mediating effects on these correlations were subsequently assessed with a partial correlation procedure for the three cognitive domains studied (Sp-A; V-Mem; Exe-F).

**Table 5:** **Correlations between direct (Wayfinding and Spatial memory scores) and self-reported (SBSOD score) navigation measures for each age group, before and after the controlling for each neurocognitive composite score (SP-A, V-Mem or Exe-F)**.

	**Correlations**		**Correlations partialled out for**			
			**SBSOD**	**SP-A**	**V-Mem**	**Exe-F**
	Wfg err	*r*	0.13	0.06	0.11	0.14
		*p*	0.47	0.74	0.57	0.43
	Wfg stop	*r*	**0.42**	**0.45**	**0.42**	**0.40**
		*p*	**0.02**	**0.01**	**0.02**	**0.02**
Young	Map	*r*	−**0.38**	−0.26	−**0.36**	−**0.34**
		*p*	**0.03**	0.15	**0.04**	**0.05**
	Pictures	*r*	−0.22	−0.24	−0.25	−0.18
		*p*	0.21	0.19	0.16	0.32
	Wfg err	*r*	0.20	0.20	0.25	0.20
		*p*	0.27	0.27	0.16	0.29
	Wfg stop	*r*	0.09	0.09	0.10	0.08
		*p*	0.63	0.63	0.60	0.64
Old	Map	*r*	−0.05	0.11	0.03	−0.04
		*p*	0.76	0.56	0.87	0.81
	Pictures	*r*	0.11	−0.04	0.05	0.11
		*p*	0.53	0.82	0.77	0.55

*Values in bold reached the significance*.

*For the young participants*, we found significant correlations between wayfinding stops and the SBSOD (*r* = 0.42; *p* = 0.02) and the Map test (*r* = −0.38; *p* = 0.03). The results from partial correlations indicated that only the Spatial abilities variable modified the relation between the Map test and the SBSOD (*r* = −0.26; *p* = 0.15). The other correlations were not significant and not modified after they were partialled out.

*For the older participants*, the correlations results did not reach the significance.

## Discussion

Very few studies have actually compared the age-related differences in navigation and spatial learning tasks according to the real vs. virtual-laboratory environment manipulation (Kalova et al., 2005; Cushman et al., 2008). Additionally, no study has yet addressed the issue of the effect of aging on the relationships between direct and self-reported navigation behavior according to the real vs. virtual-laboratory environment manipulation. Also, to our knowledge, no studies have compared the role of several cognitive mediators of age-related wayfinding and spatial learning decline in real and virtual conditions.

### Age-related differences in direct navigation measurement and their cognitive mediators according to virtual and real conditions

First, for both conditions (real and virtual), age-related differences were revealed in wayfinding performance as well as in the spatial learning tasks assessing route and survey spatial knowledge, supporting the view that aging affects the navigational processing and the acquisition processes of route and survey knowledge of large-scale spaces (Moffat, 2009; Klencklen et al., 2012). It is not excluded as noted by a reviewer that the observed age-related effect might be overexpressed by the intervisibility of distal cues across routes that is known to give more advantage for the younger adults compared to older ones (Moffat and Resnick, 2002).

Importantly, the real vs. virtual-laboratory environment manipulation has impacted the wayfinding performance without changing the observed age-related differences in both conditions. Additionally, spatial learning and age-related differences relative to route and survey knowledge measurements were not different in real and virtual conditions.

Consequently, despite the performance superiority in real-world over virtual-laboratory conditions, the magnitude of age-related differences in wayfinding performances was not significantly increased in the virtual-laboratory condition compared to the real one. This fits with the three previous studies, where a real-virtual similarity in respect of aging effect was reported for navigational behavior measures. (Kalova et al., 2005; Cushman et al., 2008; Kalia et al., 2008). Overall, the use of virtual environment tests has not artificially boosted the age differences, even if such tests have yielded lower navigation performances compared to real test conditions in young as well as in older adults. This supports the use of virtual environment testing to detect age-related declines in navigational capacities, with the limitation that virtual environment testing yields somewhat lower scores in all groups. Similarly, the age-related differences in spatial learning of naturalistic environments from VR based media mirrored almost completely those of learning resulting from direct experience. It must be nevertheless notified that our conclusions relative to survey knowledge score could be reinforced by the bidimensional regression method that probes more accurately the survey knowledge level (Tobler, 1994; Friedman and Kohler, 2003).

Taken together, these first results clearly indicated that age-related differences are significantly observed in direct navigation tests closely resembling everyday life situations. These differences are observable irrespective of the test conditions (real vs. virtual). Also, strong age differences are observed for more abstract paper-pencil tasks that explored spatial learning in real- or virtual-world test conditions. Such results are widely consistent with laboratory-based studies (e.g., Moffat, 2009), but are somewhat different of studies based on real conditions. Indeed, using real supermarket conditions, Kirasic (1991) reported that older women acquired spatial information more slowly than their younger counterparts, but failed to observe an age-related deficit in spatial cognitive performance or a benefit of environmental familiarity (see Simon et al., 1992, for similar results). Also, the study by Monacelli et al. (2003) using real hospital conditions reported strong age-differences for route learning task but no difference for map drawing tasks (albeit age-related differences were found for direction estimations). Finally, using a real hospital condition, Wilkniss et al. (1997) revealed similar results relative to wayfinding and route learning tasks than ours. Methodological differences relative to environment or task used can probably explain these discrepancies with the real-based studies. Yet, the age-related differences in navigational behaviors in novel environment reported here as in laboratory-based studies and more tenuously in real-based studies deserve to be considered as real aging outcome. Hence, it is difficult to support the view of De Beni et al. (2006) by which adequate or nearly normal performance is observable in elderly people in everyday tasks that just require recalling routes (as performed here in the wayfinding task) or self-orientation in familiar environments (see also Baroni and De Beni, 1995).

This first conclusion is reinforced by our results on the mediating effects of spatial abilities, memory and executive functioning, that are similar in both real and virtual conditions. Indeed, for the first time, we provide some empirical results favoring the idea that the age-related decline in navigation performances was mediated by both executive functioning and spatial abilities decline in both real and virtual conditions. Surprisingly, we found no mediating effect of memory decline in navigation performances, since navigation requires retrieving acquired spatial knowledge. They also seem contradictory to previous results using transfer tasks, in which memory decline is found to have a role (for review, see Moffat, 2009; Wolbers and Hegarty, 2010; Taillade et al., 2012). This discrepancy can be explained by methodological differences between the studies. Indeed, for instance, in Taillade et al. (2012) study, the relation between memory and VR-based spatial learning measure was analytically addressed by correlation analyses on raw scores from a set of three specific memory tests and from a set of four VR-based spatial learning measures. These analyses only provided four significant correlations among the 12 correlations that were tested. Thus, the relation between memory and VR-based spatial learning measures was observed for few memory measures. In other words, the mediating effect of memory decline on VR-based spatial learning performance can be considered as slight. In the present study, a composite score was computed for several memory scores. This has probably masked the slight mediating effect of memory decline on direct navigation performance.

The role of spatial abilities decline in navigation and learning in large-scale spaces is rarely examined in older adults. Previous studies in young participants showed a stronger relation between small-scale and large-scale spatial abilities in virtual than in real conditions (Hegarty et al., 2006). Our results showed a significant and similar role of spatial abilities decline in the age-related differences for navigation performances in real and virtual conditions. So, the great involvement of spatial abilities in virtual learning would not change despite the importance of their influence on the age-related decline of spatial performance.

Concerning the role of memory and executive declines, our result could be consistent with some studies using cerebral imagery (Moffat et al., 2006; Antonova et al., 2009) where sometimes the neuronal circuit of executive functioning (i.e., prefrontal structure and caudate nucleus) is demonstrated as more related to navigation performances in both young and old participants than that of memory functioning (i.e., hippocampal structure), which suggests a stronger role of executive rather than memory functioning in age-related differences on navigational behaviors (Moffat, 2009; Klencklen et al., 2012).

To recapitulate, similar age-related differences are observed in real and virtual conditions. And, spatial abilities and executive functioning are reported as cognitive mediators of age differences observed on navigation performance, irrespective of virtual or real conditions. This supports the ecological validity of VR applications in assessing the effect of aging on navigation performance.

### Age effect on self-reported navigation performance

The older adults had similar scores to young adults on the SBSOD, which provides self-reported measurements of everyday navigation. This is in accordance with previous studies (Kirasic et al., 1992; Baroni and De Beni, 1995; Burns, 1999; De Beni et al., 2006; Taillade et al., 2012, 2013; Borella et al., 2014) that have used different self-reported navigation measurement methods. So, the absence of age difference in self-reported navigation performance is consistent across the studies and across the various self-reported navigation scales. Conclude that there is a metacognitive decline with age is still premature, given that earlier results showed real but minor age differences in real-world navigation tests. Indeed, if age-related changes in navigation are minor in everyday situations, they could be more difficult to detect, rendering their monitoring and self-awareness more difficult.

In summary, although actual age differences are reported as directly measurable in relation with navigation behavior, elderly adults did not differ from young adults in their self-reporting of everyday navigation, suggesting some underestimation of navigation difficulties by elderly adults. To elucidate the issue of possible age changes in the awareness of everyday navigation functioning, we performed correlation analyses to capture relationships between direct and self-reported measures of large-scale spatial abilities, and also their relationships with small-scale spatial abilities, memory, and executive functioning.

### Relationships between direct and self-reported navigation performances according to age conditions

When the composite scores of navigation performance are considered (ZNav score), correlations with self-reported performance (SBSOD score) were not found. This lack of relation can be explained by previous observations in young participants showing that self-reported sense of direction (as the SBSOD score) are more related to direct navigation performances or survey knowledge-based scores than to route knowledge-based scores (e.g., Hegarty et al., 2002). In accordance with these observations, we found significant correlations between self-reported and objective navigation performances for specific navigation and survey knowledge-based measures (the wayfinding stops and the map drawing score), but only for young participants. When we controlled for spatial abilities, correlations between self-reported and objective performances were no longer significant. These results are consistent with those of Hegarty et al. (2006), who found that the SBSOD was a predictor of environmental learning, after the effect of spatial abilities was controlled. We can also remark that the SBSOD is considered as a measure of everyday difficulties for learning and navigating in large-scale spaces, but also a measure of spatial updating performances which is also strongly related to learning performances in large-scale spaces.

For older adults, no significant correlation was obtained even when spatial abilities were considered. The age-related loss of relations between self-reported navigation performances and spatial abilities may provide a relevant explanation of the loss with age of relations between direct and self-perceived navigation performance. As seen before, the age differences on direct navigation performance are mostly explained by the decline in spatial abilities due to aging. Consequently, if the self-estimates of older people do not rely on their spatial abilities, such self-estimates may be distorted and may be also biased by older people's beliefs about their own navigation skills, which thus supports metacognitive difficulties in older adults (Hertzog, 2002). There is growing evidence that age stereotypes influence self-judgment in the elderly and is domain-specific (e.g., Kite et al., 2005; Kornadt and Rothermund, 2011). For instance, the older adults have a negative judgment of age on memory functioning (Hess et al., 2003) and neutral or even positive judgment on their spatial abilities (Lawton, 1994). Also, like in other cognitive domains such as memory, several hypotheses can be advanced to explain the lack of a relation to aging between self-reported and objective performances. These hypotheses could be based on mood, self-esteem, experience level (Volz-Sidiropoulou and Gauggel, 2012). For instance, older adults might base their navigation estimates on past-succeeded experiences of which they have a broader range than younger people. Thus, they might underestimate their current navigation difficulties. Such assumption has its importance in the present study since we used a global self-reported navigation measure (SBSOD score) that probably inflates the self-estimate biases in old participants compared to specific self-reported measures or self-estimates of expected performance focusing on wayfinding or spatial memory tasks. Also, the lack of significant correlations between navigation composite scores and self-reported performances, irrespective of age, is supportive of this assumption. Consequently, this stresses that the metacognition-related explanations deserve a deep examination in further studies where the navigation performance should be detailed in their actual and self-reported facets.

## Conclusion

Our results showed comparable effects of age on large-scale spatial abilities in real and virtual performances, confirming the ecological validity of the assessment of wayfinding performances with virtual reality. Importantly, for the first time, it has been demonstrated that the cognitive mediators of navigation performances in real and virtual conditions were similar, since we found that age-related navigation difficulties were dependent on both executive and spatial abilities. Such age-related difficulties might probably decrease with routes performed without intervisibility (i.e., proximal cues are only available for navigation). Indeed, the distal cues might play a major role in the age-related differences observed on navigation performances. This means that the age-related differences reported here might not necessarily generalize to other environments (those without intervisibility). Further studies should be done for specially addressing this issue.

Regarding self-reported difficulties, spatial abilities in the young participants played a mediating role in the relationship between objective and subjective navigation performance, but not for the older ones. Thus, the relationship between objective and subjective measures of navigation performances is actually observed in young participants but not for older adults. This loss of relationship with age should be better endorsed by further investigations with specific self-reported navigation measures because, as mentioned above, our general self-reported measure (i.e., SBSOD score) is probably a limitation for capturing the relationships between actual and self-reported performances, particularly when they refer to route-knowledge.

To sum up, the present results support the ecological validity of virtual applications for the assessment of age-related changes in navigational behaviors. The lack of relation between objective and subjective navigation measures only for older adults reinforces the use of direct measures for older adults. Taken together, our results raise the importance of examining in detail the various reasons responsible for the loss with age of relations between self-reported cognitive ability and test performance in the domain of navigation.

### Conflict of interest statement

The authors declare that the research was conducted in the absence of any commercial or financial relationships that could be construed as a potential conflict of interest.

## Footnotes

^1^French law in Public Health (08/08/2004, ≪ article L.1121-5 ≫).

^2^This VE was a realistic environment in term of visual rendering but it was not an analog of a real environment.

^3^As mentioned by a reviewer, the route to-be learn exhibits some intervisibility, particularly in intersection parts.
